# Results from extended lymphadenectomies with [^111^In]PSMA-617 for intraoperative detection of PSMA-PET/CT-positive nodal metastatic prostate cancer

**DOI:** 10.1186/s13550-020-0598-2

**Published:** 2020-03-06

**Authors:** Cordula A. Jilg, Kathrin Reichel, Christian Stoykow, H. Christian Rischke, Mark Bartholomä, Vanessa Drendel, Moritz von Büren, Wolfgang Schultze-Seemann, Philipp T. Meyer, Michael Mix

**Affiliations:** 1 Department of Urology, Medical Centre – University of Freiburg, Faculty of Medicine, University of Freiburg, Hugstetterstraße 55, 79106 Freiburg, Germany; 2 Department of Nuclear Medicine, Medical Centre – University of Freiburg, Faculty of Medicine, University of Freiburg, Freiburg, Germany; 3 Department of Radiation Oncology, Medical Centre – University of Freiburg, Faculty of Medicine, University of Freiburg, Freiburg, Germany; 4 Institute for Pathology, Faculty of Medicine, University of Freiburg, Freiburg, Germany; 5 German Cancer Consortium (DKTK), Partner Site Freiburg, Freiburg, Germany; 6 Department of Medical Imaging and Clinical Oncology, Nuclear Medicine Division, Faculty of Medicine and Health Science, Stellenbosch University, Stellenbosch, South Africa

**Keywords:** Radio-guided surgery, [^111^In]PSMA, Lymphadenectomy, Prostate cancer, Salvage lymph node dissection

## Abstract

**Purpose:**

Identification of suspicious PSMA-PET/CT-positive lymph node (LN) metastases (LNM) from prostate cancer (PCa) during lymphadenectomy (LA) is challenging. We evaluated an ^111^In-labelled PSMA ligand (DKFZ-617, referred to as [^111^In]PSMA-617) as a γ-emitting tracer for intraoperative γ-probe application for resected tissue samples in PCa patients. Forty-eight hours prior to LA, [^111^In]PSMA-617 was administered intravenously in 23 patients with suspected LNM on PSMA-PET/CT (*n* = 21 with biochemical relapse, *n* = 2 at primary therapy). Resected tissue samples (LN, LNM and fibrofatty tissue) were measured ex situ by a γ-probe expressed as counts per second (CPS_norm_). [^111^In]PSMA-617 tissue sample uptake was measured by a germanium detector for verification and calculated as %IA_lbm_ (percent injected activity per kilogram lean body mass at time of surgery). Based on a clinical requirement for a specificity > 95%, thresholds for both ex situ measurements were chosen accordingly. Correlation of the results from PET/CT, γ-probe and germanium detector with histopathology was done.

**Results:**

Eight hundred sixty-four LNs (197 LNM) were removed from 275 subregions in 23 patients, on average 8.6 ± 14.9 LNM per patient. One hundred four of 275 tissue samples showed cancer. Median γ-probe and germanium detector results were significantly different between tumour-affected (33.5 CPS_norm_, 0.71 %IA_lbm_) and tumour-free subregions (3.0 CPS_norm_, 0.03 %IA_lbm_) (each *p* value < 0.0001). For the chosen γ-probe cut-off (CPS_norm_ > 23) and germanium detector cut-off (%IA_lbm_ > 0.27), 64 and 74 true-positive and 158 true-negative samples for both measurements were identified. Thirty-nine and 30 false-negative and 6 and 5 false-positive tissue samples were identified by γ-probe and germanium detector measurements.

**Conclusion:**

[^111^In]PSMA-617 application for LA is feasible in terms of an intraoperative real-time measurement with a γ-probe for detection of tumour-affected tissue samples. γ-probe results can be confirmed by precise germanium detector measurements and were significantly different between tumour-affected and tumour-free samples.

## Background

Prostate cancer (PCa) is the most commonly diagnosed cancer in men. It can be cured by surgical removal of the prostate (radical prostatectomy) with pelvic lymphadenectomy (LA) or by radiotherapy (RT) [1–4]. The risk of metastases is mainly determined by the Gleason score, the level of PSA (prostate-specific antigen) and tumour extension (TNM status) [3, 4]. Pelvic and retroperitoneal lymph nodes (LN) are the first sites for metastases [2–4]. LN metastases (LNM), if assumed at primary therapy, should be removed at radical prostatectomy with LA in order to improve oncological outcome and enable correct staging [2–5]. An accurate preoperative detection of LNM by, e.g., positron emission tomography/computed tomography (PET/CT) is a prerequisite for successful surgery [6, 7].

Despite primary therapy, about 15–30% of the patients will develop a biochemical recurrence with elevated PSA level and clinical recurrence (metastases) possible at different sites (e.g. local relapse, LNM, bone). A systemic therapy such as ADT or chemotherapy is still the standard treatment in the stage of metastatic prostate cancer [3]. Regretfully, during systemic therapy, PCa will inevitably develop a resistance to androgen deprivation or chemotherapy, which will end up in tumour progression again.

PET/CT targeting prostate-specific membrane antigen (PSMA) has demonstrated an excellent ability to detect LNM prior to surgery [6, 8–11] and is widely used as a tool for staging before primary therapy restaging of PCa patients in the setting of biochemical relapse [8–10, 12].

The most widely studied is ^68^Ga labelled to the small molecular inhibitor PSMA-11 via the HBED chelator ([^68^Ga]PSMA-11, also known as [^68^Ga]PSMA-HBED-CC) [13]. A recent meta-analysis published by Perera et al. showed on a per lymph node analysis a high sensitivity of 75% and a 99% specificity [14] for [^68^Ga]PSMA-11 PET/CT. Because of several advantages of the positron emitter ^18^F, ^18^F-labelled PSMA ligands ([^18^F]PSMA-1007, [^18^F]DCFPYL) were used more and more for imaging instead of ^68^Ga-labelled PSMA ligands [15, 16].

If PET/CT indicates “regional pelvic LNM” as the only finding at clinical recurrence, surgical removal (i.e. “salvage lymphadenectomy” (salvage-LA [17, 18]) of the lymphatic tissue or targeted RT may be suggested in patients in good general condition [6, 18, 19]. These “active approaches” are offered in order to delay systemic therapies [3, 18, 20, 21]. Against the background of the guidelines (e.g. European guidelines), salvage LND or target RT should be considered to be experimental and individual therapeutic approaches [3].

Locating suspected recurrent LNM during surgery is often very challenging in case of small LNM and reduced accessibility to the LNM (e.g. because of atypical location of LNM and tissue adhesions) [11]. Positron-emitting tracers are highly suitable for PET imaging but inappropriate for locating LNM during surgery because the γ-energy of their annihilation photons is too high for standard γ-probe devices. More appropriate for tracking of radioactively tagged LNM during surgery are γ-emitting tracers such as [^111^In]PSMA-I&T [22], [^99m^Tc]PMSA-I&S [23] or [^111^In]PSMA-DKFZ-617 [24] which also allow imaging as the PET tracers but with reduced spatial resolution, sensitivity and contrast due to the single photon emission computer tomography (SPECT) imaging technique [11, 25–27]. Without any doubt, there is a clear need to improve the identification of LNM during difficult surgery, regardless if at primary LA or at salvage LA [6, 11, 26, 28].

^111^In-labelled PSMA ligands were successfully introduced recently for imaging as well as for intraoperative use (radio-guided surgery (RGS)) [11, 21, 28–30]. After intravenous tracer injection prior to surgery, suspected LNM could be identified during surgery by applying a γ-probe with acoustic feedback. Accordingly, the surgeon is able to conduct in situ and ex situ measurement of suspected regions for LNM and resected tissue samples.

The recent development in the field of RGS for nodal recurrent PCa relapse is the application of ^99m^Tc-labelled PSMA by Maurer et al. [25]. Based on the investigation of 31 patients with nodal PCa recurrence undergoing a salvage LND with the use of a γ-probe, they could show (specimen based) an impressive sensitivity of 83.6%, a specificity of 100% and an accuracy of 93% [25]. However, the identification of an optimal tracer for RGS salvage LND is still under investigation [26], and RGS is applied differently at the institutions offering this kind of experimental approach [11, 26, 31].

Recently, our group investigated the tracer uptake of [^111^In]PSMA-617 at a “single LN level” (manual separation of the resected tissue samples into single LN and LNM) in six patients demonstrating an excellent performance for the distinction between affected and non-affected LN (92.1% sensitivity, 98.9% specificity) [11].

The aim of this report was to evaluate the performance of the [^111^In]PSMA-617 on “region level” for a larger number of cases by analysing resected tissue samples from LA in order to distinguish between affected (mainly LNM) and non-affected tissue in men with suspected LNM on a PSMA-PET/CT using [^68^Ga]PSMA-11. Counts per second measured by γ-probe (ex situ), tissue sample tracer uptake measured with a germanium detector (ex situ) and PET/CT findings were correlated with histopathology. Based on the clinical requirement for high specificity (e.g. > 95%), the thresholds for both ex situ measurements were selected accordingly.

## Material and methods

### Patients

Between May 2015 and October 2016, 23 patients with the suspicion of exclusive LNM (without detectable bone or visceral metastases) on PSMA-PET/CT underwent LA guided by [^111^In]PSMA-617. Two of 23 patients underwent extended LA in the primary setting (radical prostatectomy), and 21/23 patients at the stage of biochemical recurrence (PSA > 0.2 ng/ml after radical prostatectomy) underwent salvage LA on a compassionate use basis. The local ethics committee approved this retrospective data analysis (no. 562/15). Informed consent was obtained from each subject, and all procedures were performed in accordance with the Helsinki Declaration. After surgery, the patients were treated according to the German S3 guidelines for treatment of PCa [2]; in case of PCa progression, a restaging by imaging (preferable PSMA-PET/CT) was done.

Tissue samples from eight subregions were excluded from the contingency analysis because a follow-up PSMA-PET/CT clearly showed the persistence or progression of the PET-positive lesion(s) and thereby that the LN(s) had obviously not been removed during surgery (e.g. difficulty in surgical access or because of inappropriate high surgical risks). Consistently, tissue samples from these “true-PET-positive” subregions have been therefore excluded from the analysis due to absence of tumour tissue and correspondingly negative γ-probe and germanium detector measurements.

Mean PSA follow-up was 31.2 ± SD 12.8 months; median follow-up at latest restaging (imaging) was 25.7 ± SD 16.3 months.

### PSMA-HBED-CC-PET/CT and imaging analysis

[^68^Ga]PSMA-11 was conducted as described by Jilg et al. [6, 11]. Imaging was done 1 h after injection of averaged 202 ± SD 25 MBq [^68^Ga]PSMA-11. Contrast-enhanced diagnostic CT was used for anatomical correlation and PET attenuation correction. Two experienced nuclear medicine physicians evaluated all the PET/CT studies in consensus by side-by-side review of the co-registered PET and CT datasets using predefined PET window settings (inverted grey scale, SUV range 0 to 5 g/ml). All patients showed increased focal [^68^Ga]PSMA-11 uptake in at least one pelvic and retroperitoneal region. A PSMA-positive lesion was defined as focal tracer accumulation at least two times greater than normal or physiological local background activity.

### Synthesis of ^111^indium-labelled PSMA

The DOTA (1,4,7,10-tetraazacyclododecane-1,4,7,10-tetraacetic acid)-conjugated PSMA-617 was labelled with ^111^InCl_3_ under Good Laboratory Practice within 30 min at 95 °C in ammonium acetate buffer. PSMA-617 was obtained from ABX advanced biochemical compounds (Radeberg, Germany). The radiotracer solution was prepared by dilution with 0.9% NaCl. The radiochemical purity of the final product was ≥ 97% and the decay-corrected yield was > 95%. On average 44 ± 10 h prior to surgery, patients received an intravenous application of [^111^In]PSMA-617 (mean 110 ± 14 MBq). A PSMA single-photon emission CT (SPECT/CT) was conducted with a mean of 23.4 ± 1.2 h prior to surgery to ensure sufficient tracer accumulation in the suspected tumour lesions.

### Procedure at lymphadenectomy

The extent of LA was determined first by the aim to adhere to a template LA according to the presence of PET-positive lesions: in the case of a pelvic PET-positive lesion(s), a bilateral pelvic LA was intended whenever possible (conducted in 19/23 men). In the case of an additional PET-positive lesion in the retroperitoneum, a retroperitoneal LA was conducted (Table 1). Subregions for a template pelvic bilateral LA were as follows: common iliac vessels, external iliac vessels, obturator vessels, internal iliac vessels (presacral region). Subregions for a template retroperitoneal LA were as follows: aortic bifurcation, aortal, caval, as described by Jilg et al. [6]. Whenever permitted by the intraoperative circumstances (deviation from the template, e.g. caused by surgical difficulties), we adhered to this template. Nodal fibrofatty tissue from each subregion was collected separately at surgery [6]. Furthermore, the knowledge of the PET/CT results (location of suspected LNM) also had an impact on the extent of LA in the corresponding subregion(s). Finally, the number of counts from the γ-probe used intraoperatively (ex situ measurement) gave the surgeon feedback if the suspected tumour tissue was resected or if the LA in this subregion had to be continued which ultimately increased the intensity of LA in this subregion.

**Table 1:** Patient characteristics, history of prostate cancer and outcome from lymphadenectomies from 23 patients undergoing lymphadenectomy

Parameters	Values
iPSA at primary therapy (ng/ml)	
Mean ± SD/median/range	10.79 ± 7.5/8.8/3.37–37.0
Primary therapy, *n*	
Radical prostatectomy	21/23 (91.3%)
Radiotherapy	2/23 (8.7%)
Gleason score, *n*	
7a	4 (17%)
7b	7 (31%)
8	5 (22%)
9	7 (30%)
^111^In-PSMA-guided LA overall, *n*	
Primary	2/23 (8.7%)
Salvage lymph node dissection	21/23 (91.3%)
Age at lymphadenectomy (years)	
Mean ± SD/median/range	67.5 ± 6.6/67/52–78
PSA at ^111^In-PSMA-guided LA (ng/ml)	
Mean ± SD/median/range	7.9 ± 12.9/1.8/0.03–56.2
Time between PET/CT and ^111^In-PSMA-guided LA (months)	
Mean ± SD/median/range	3.2 ± 1.6/3.0/1.0–8.0
Time between primary therapy and ^111^In-PSMA-guided LA (years) (*n* = 21)	
Mean ± SD/median/range	4.9 ± 3.7/4.4/1.5–13.7
Histological outcome for 23 patients, *n*	
LA with positive histology	21/23 (91.3%)
LA with negative histology	2/23 (8.7%)
Topography of ^111^In-PSMA-guided LA in 23 patients, *n*	
Pelvic right and left	13/23 (57%)
Pelvic left only	2/23 (9%)
Pelvic right only	1/23 (4%)
Pelvic right and left and retroperitoneal	7/23 (30%)
Topography of subregions with confirmed PCa, *n*	
Pelvic left, *n*	38/275 (37%)
Pelvic right, *n*	41/275 (40%)
Retroperitoneal	24/275 (23%)
Histological outcome for 275 subregions with 275 samples, *n*	
Subregions/samples with LNM	104/275 (37.5%)
Subregions/samples without LNM	171/275 (62.5%)
Subregions/samples with additional non-nodal PCa-tissue	7/104 (5.8%)
Number of LN removed (*n*)	
Overall	864
Per patient (mean ± SD/median/range)	37.6 ± 17.2/38.0/2.0–82.0
Number of LNM removed (*n*)	
Overall	197
Per patient (mean ± SD/median/range)	8.6 ± 14.9/4.0/0.0–71.0
LNM fraction per patient (LNM×100/LN = %)	
Mean ± SD/median/range	20.9 ± 24.4/12.5/0.0–87.1

*LNM* lymph node metastases, *LN* lymph node, *PSA* prostate-specific antigen, *LA* lymphadenectomy

### Analysis with γ-probe

After removal of the 275 specimens, counts per second (CPS) were registered with a γ-probe (Neoprobe® GDS ex situ). To generate comparable data between patients, CPS were normalised (CPS_norm_) to the injected activity per kilogram lean body mass and decay-corrected to the time of surgery (48 h representing the median time after injection of [^111^In]PSMA-617 and the median time of ex situ γ-probe measurements) in the patient group.

### Analysis with germanium detector

All samples were weighed. Tissue sample activity measurements were done with a high-purity germanium detector (Canberra Inc., model GX2018-CP5+, calibrated with a multi-isotope reference source, type VZ-2139/NG3 from Eckert&Ziegler Nuclitec’s DKD-accredited measurement laboratory in Germany, and cross-calibrated for tissue sample geometry). Tracer uptake was calculated as percent injected activity per kilogram lean body mass, corrected for decay:

$$ \%\mathrm{IAlbm}=\frac{\mathrm{tissue}\ \mathrm{sample}\ \mathrm{activity}\ \left[\mathrm{Bq}\right]\cdot 100}{\left(\mathrm{injected}\ \mathrm{activity}\ \left[\mathrm{Bq}\right]\bullet {2}^{-\Delta  \mathrm{t}/{\mathrm{T}}_{1/2}}\right)/\mathrm{lean}\ \mathrm{body}\ \mathrm{mass}\ \left[\mathrm{kg}\right]} $$

with Δ*t* and *T*_1/2_ being the delay between patient injection and sample measurement and the half-life of ^111^In (2.81 days [32]), respectively. Lean body mass was calculated according to Janmahasatian et al. [33] and used for normalisation instead of body weight, since fat tissue does not participate in normal tracer distribution.

### Histopathological analysis

All resected LNs (i.e. the entire LN in case of small LNs, one central slice in case of LNs > 4 mm) were fixed with formalin and embedded in paraffin. The pathologist was not aware of the PET findings and did not know the clinical report of the tissue from the surgeon. Histopathologic evaluation was performed by one pathologist on haematoxylin and eosin (H&E)-stained tissue slides.

### Statistical analysis

Analysis of findings (positive, negative) of PET/CT, **γ**-probe and germanium detector in a contingency table was done for 267 of 275 subregions; 8 subregions were excluded from the contingency analysis because a follow-up PSMA-PET/CT showed the persistence or progression of the PET-positive lesion(s) indicating that the LN(s) had not been removed during surgery. Descriptive statistics were done by calculating means, standard deviations (SD), medians and ranges. From all resected tissue samples, we had the information of the γ-probe and germanium detector results as well as the histopathological outcome. Based on these data, we performed a ROC curve analysis (*receiver operating characteristic curve)*. Continuous variables were compared with a two-sided unpaired Mann-Whitney test. Diagnostic accuracy of γ-probe (CPS_norm_), germanium detector tracer-uptake measurement (%IA_lbm_) and PET/CT was described by a contingency table. We used Prism 6 GraphPad.

## Results

Clinical data summarising the stage of PCa and LAs of the 23 patients are shown in Table 1. In the majority (21/23), LA were done in the setting of salvage LA because of suspected LNM on a PSMA-PET/CT at the stage of biochemical recurrence. Mean PSA level at surgery was 7.9 ng/ml. For 21 of 23 patients, histological outcome confirmed the presence of PCa in the removed tissue samples; in the remaining 2/23 patients, it was not possible to remove the PET-positive lesions. By PSMA-PET/CT follow-up, the PET-positive lesions in those two patients showed a clear progression or persistence of the metastases. Complications from surgery, analysed according to Clavien-Dindo [34], are shown in Table 2.

**Fig. 1:**
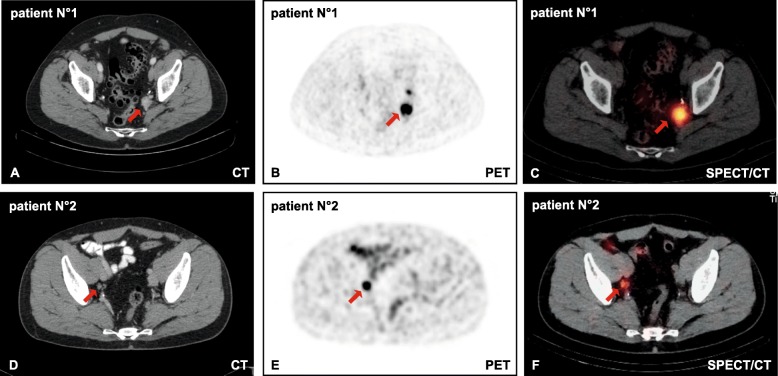
**a**, **d** Transversal CT and **b**, **e** PET of a PSMA-PET/CT with from two different representative patients no. 1 and no. 2 with suspected lymph node metastases (LNM) prior to lymphadenectomy. **c**, **f** Preoperative (48 h preoperatively) transversal SPECT of the same patient. Suspected LNMs are indicated by a red arrow

Figure 1a–f shows two representative PSMA-PET/CT and SPECT/CT with an LNM in the left parailiacal region (patient no. 1) and in the right parailiacal region (patient no. 2). A median of 38 LN had been removed during LA per patient, of which a median of 4.0 turned out to be metastases. Overall, a high number of subregions (*n* = 275) underwent LA, and a high number of LN (*n* = 864) had been removed (Table 1).

**Fig. 2:**
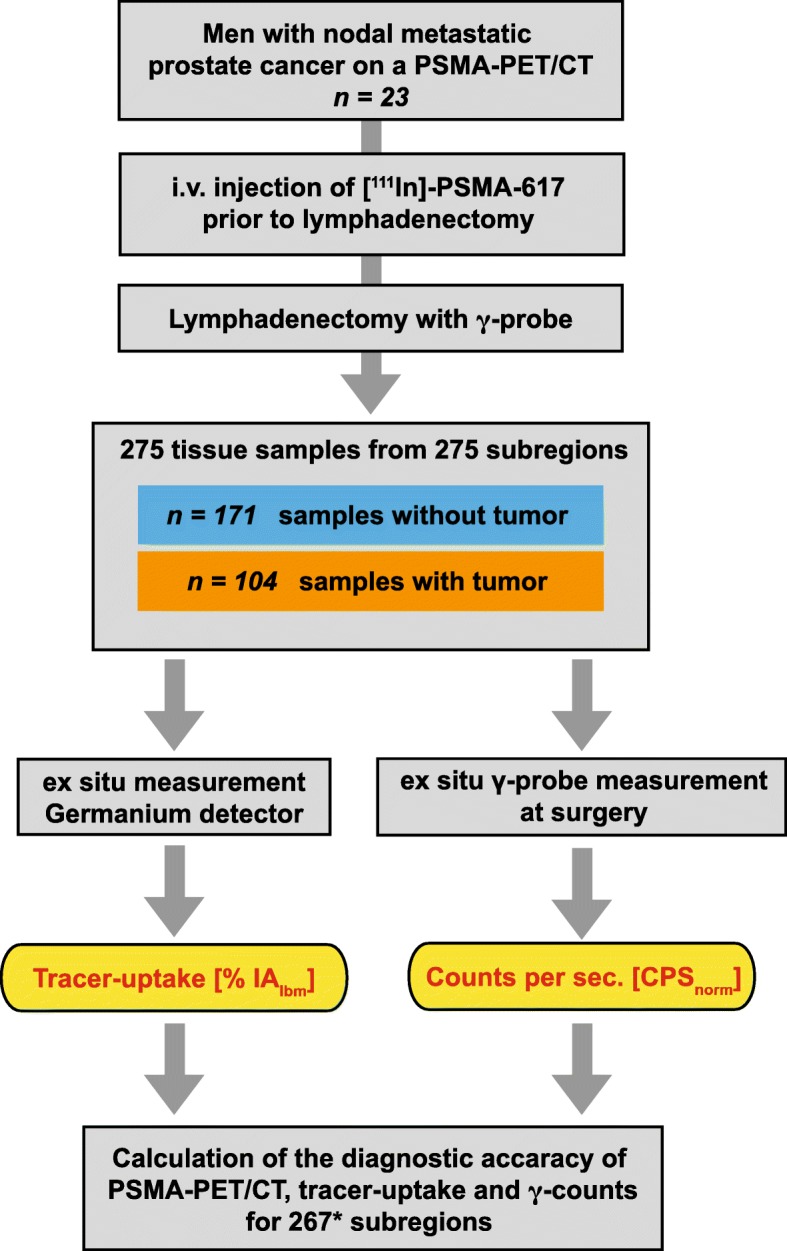
Workflow and tissue sample processing from 23 lymphadenectomies after surgery with [^111^In]PSMA-617. Resected tissue specimens (consisting out of LN, LNM and fibrofatty tissue) from a total of 275 subregions were analysed with a γ-probe at surgery followed by histopathological analysis. Tracer uptake in tissue samples was analysed with a germanium detector. The asterisk symbol indicates that 8 of 275 subregions were excluded from the analysis because a follow-up PSMA-PET/CT showed the persistence or progression of the PET-positive lesion(s) indicating that the LN(s) had not been removed during surgery

The workflow of the sample processing is shown in Fig. 2. From 275 subregions, 275 tissue samples, consisting of LN, LNM and fibrofatty tissue, were removed separately and measured with a γ-probe and in a germanium detector ex situ. The origin of 275 tissue samples is shown in Table 1.

**Fig. 3:**
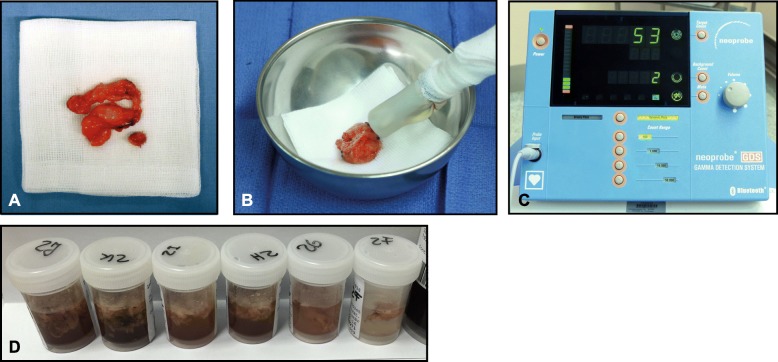
**a** Representative resected tissue sample from a subregion consisting of lymph nodes and fibrofatty tissue. **b**, **c** γ-probe measurement (counts per second) from a tissue sample. **d** Representative collection of 6 tissue samples from 6 subregions for analysis of tracer uptake in the germanium detector

Figure 3 shows representative nodal fibrofatty tissue samples from one subregion (a, b), γ-probe measurements (c) and sample vessels for the tracer uptake measurements (d).

Histopathological analysis of the 275 tissue samples yielded 171 samples free of tumour and 104 samples with tumour (mainly LNM).

**Fig. 4:**
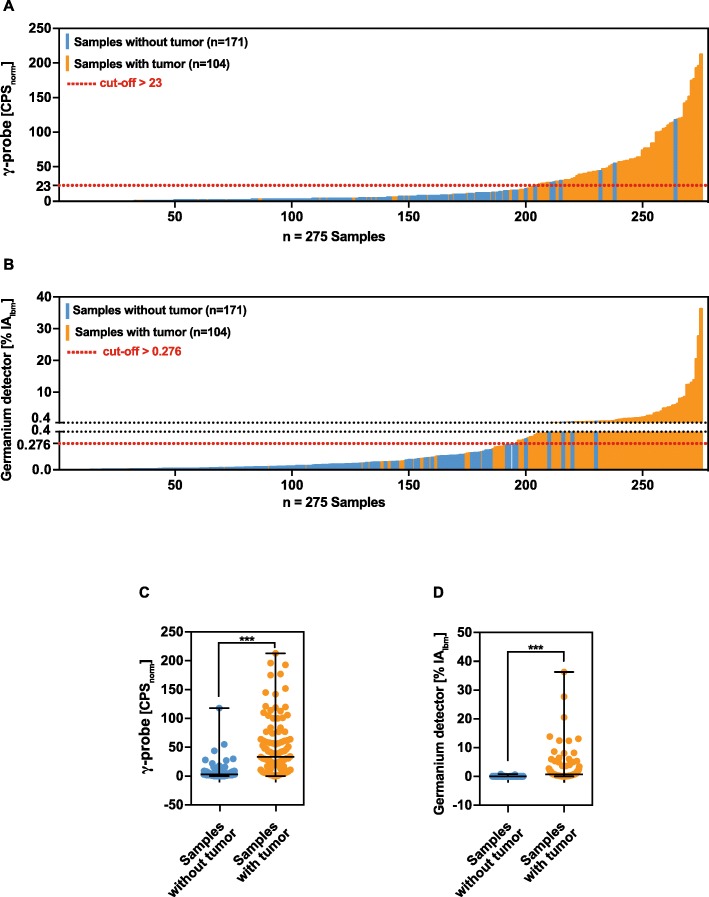
**a** Waterfall plot of the CPS_norm_ [CPS/(MBq/kg)] and **b** tracer uptake [%IA_Ibm_] of tumour-free samples and tumour-containing samples illustrating the power of discrimination of [^111^In]PSMA-617. CPS_norm_ (**c**) and %IA_Ibm_ (**d**) from samples with and without tumour is shown in scatter plots; horizontal black lines represent the medians ± range. ***p ≤ 0.001

Results of CPS_norm_ and %IA_lbm_ tracer uptake from tumour-containing (*n* = 104) and tumour-free (*n* = 171) samples were significantly different (both *p* < 0.0001) which is shown in Table 3. Samples without tumour had a median of 3.0 CPS_norm_ and 0.03 %IA_lbm_ compared with a median of 33.5 CPS_norm_ and 0.71 %IA_lbm_ measured from samples with tumour. This high degree of separation by γ-probe and germanium detector measurements is also shown in Fig. 4 c and d by comparing the medians ± range of CPS_norm_ and %IA_lbm_ in scatter plots. Each corresponding value of CPS_norm_ and %IA_lbm_ from 275 tissue samples is displayed in Fig. 4 a and b in a waterfall plot. Red lines highlight the cut-off(s) for both measurements.

**Table 2:** Complications arising from 23 lymphadenectomies

Complications according to Clavien-Dindo	Grade	Overall, *n* (%)
Lymphorrhea	I	3/23 (13.04%)
Transient weakness of hip flexor	I	1/23 (4.35%)
Wound dehiscence	I	1/23 (4.35%)
Wound infection treated with antibiotics	II	1/23 (4.35%)
Lymphorrhea requiring drainage, secondary	IIIa	2/23 (8.7%)

Based on the clinical requirement of high specificity (> 95%), a threshold of > 23 CPS_norm_ and > 0.27 %IA_lbm_ was determined to reduce the risk of false-positive samples. For a cut-off of > 23 CPS_norm_, we determined a sensitivity of 62.1% and specificity of 96.3%. It has to be mentioned that for patient-individual γ-probe CPS measurements during surgery, the threshold of 23 CPS_norm_ has to be multiplied by the injected activity per kilogram lean body mass. After applying the cut-off of > 0.27 %IA_lbm_, we calculated a sensitivity of 71.2% and a specificity of 96.9% comparable to PET/CT (sensitivity of 79.8% and specificity of 96.9%) (Table 4).

**Table 3:** γ-probe and germanium detector measurements from 275 tissue samples of 275 subregions

	Samples with tumour (*n* = 104)	Samples without tumour (*n* = 171)	Mann-Whitney test, *p* value
Germanium detector [% IA_lbm_]*			
Median/mean ± SD/range	0.71/2.6 ± 5.4/0.008–36.33	0.03/0.07 ± 0.17/0–0.817	< 0.0001
γ-probe [CPS_norm_]^†^			
Median/mean ± SD/range	33.5/49.0 ± 49.5/0–213.0	3.0/5.9 ± 11.2/0–118.0	< 0.0001

^*^Percent injected activity per kilogram lean body mass

^†^Counts per second decay corrected to 48 h and normalised to injected activity megabecquerel per kilogram lean body mass

**Fig. 5:**
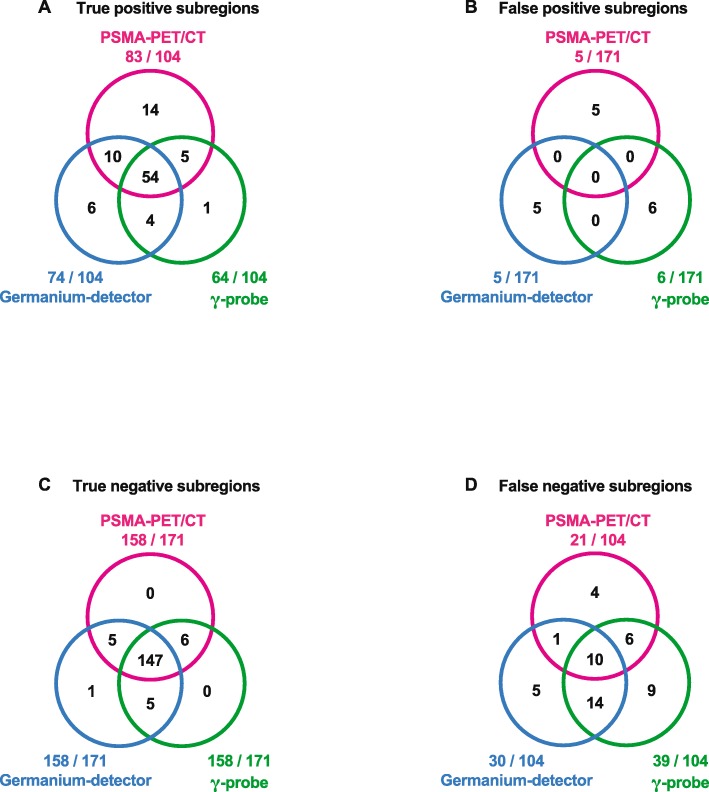
**a**–**d** Venn diagrams illustrating the intersections between the results of PSMA-PET/CT, tracer uptake determined with the germanium detector and ex situ γ-probe measurements. Intersections for **a** true-positive, **b** false-negative, **c** true-negative and **d** false-positive subregions in 104 subregions with histologically proven metastases and 171 subregions free of metastases

Figure 5 shows the intersections of true- and false-positive as well as true- and false-negative subregions from PSMA-PET/CT findings, γ-probe and germanium detector measurements with Venn diagrams.

One hundred forty-seven of 171 (85.9%) specimens free of cancer were correctly identified as true negative by all three modalities (Fig. 5c). In contrast, only 54/104 (51.9%) of the specimens with cancer had been identified correctly as true positive by all three modalities (Fig. 5a).

Additional file 1: Table S1 shows data about the clinical course after surgery regarding PSA values and follow-up imaging by mainly PSMA-PET/CT. Most (14/23) of the patients developed clinical progression after salvage LND.

## Discussion

By targeting PSMA on cancer cells with ^111^In- or ^99m^Tc-labelled PSMA ligands, RGS was successfully introduced for intraoperative use with a γ-probe and acoustic feedback by Maurer et al. [11, 21, 25, 26, 28]. As those previous works analysed so-called “mixed-tissue samples” containing LN, LNM and fibrofatty tissue, a more specific investigation of the [^111^In]PSMA-617 tracer uptake at a single LN level was done by our group [11]. Based on 275 tumour-free single LNs and 35 single LNMs, after manual separation out of resected tissue samples, a sensitivity of 92.1% and specificity of 98.9% were reported, indicating a convincing tumour-specific uptake in PCa lesions [11].

Rauscher et al. reported on 31 men undergoing RGS with ^111^In-labelled PSMA ligand because of nodal PCa relapse. One hundred forty-five mixed-tissue samples were resected (51 tumour bearing) and measured ex situ with a γ-probe. Sensitivity was 92.3%; specificity was 93.5% [29]. RGS described by Maurer et al. and Rauscher et al. was predominantly restricted to PET-positive regions only and their immediate vicinity [28, 29].

A unique feature in our study is the measurement of tracer uptake of the resected tissue samples directly after surgery in a germanium detector, which can be considered as a precise verification of γ-probe measurement. Such an effort is not a part of clinical routine and was done only for the cohort presented here. Due to tracer availability, isotope costs and radiation protection issues, ^99m^Tc-labelled PSMA is currently used for RGS, and our cohort provides uniform data from the relatively limited experience with RGS ^111^In-labelled PSMA.

Although in situ γ-probe measurement was done in this study, a usual standard γ-probe with “pencil-geometry” is not well engineered yet in terms of size and angle for the application in a narrow pre-treated pelvic region.

During practice in salvage LND with a γ-probe, it turned out that sufficient contact between the γ-probe and the tissue region (ideally 90° corresponding to the collimator angle) in situ was not always possible because of restricted spatial conditions. Therefore, we focused on the ex situ measurements of the removed tissue samples with the γ-probe (CPS_norm_) and the subsequent tracer uptake measurements with a germanium detector (%IA_lbm_).

Usually, tissue samples from one subregion consist of LN, LNM, fat, small vessels and fibrofatty tissue. Because of unspecific tracer uptake within such a tissue mix, false-positive signals might arise at γ-probe and germanium detector measurements. On the other hand, a specific tracer signal in tumour tissue might be weakened due to attenuation in large mixed-tissue samples enclosing fibrofatty tissue, fat or small vessels, leading to false-negative results, especially in the case of a very small amount of tumour tissue.

Because of the clinical need for a low number of false-positive results (to prevent the surgeon from misinterpretation and early termination of the LA), expressed by a high specificity (e.g. > 95%), we chose the threshold for both measurements appropriately. Consequently, both measurements yield a relatively low sensitivity (Table 4).

**Table 4:** Agreement between PSMA-PET/CT, γ-probe, germanium detector and histopathology in 267 subregions

Subregions with tissue samples (*n* = 267*)		Histopathology		Sensitivity (%)	Specificity (%)	PPV (%)	NPV (%)
		Positive	Negative				
PSMA-PET/CT findings^†^	Positive	83	5	79.8	96.9	94.3	88.3
	Negative	21	158				
γ-probe [CPS_norm_]^‡^	Positive	64	6	62.1	96.3	91.4	80.2
	Negative	39	158				
Germanium detector [% IA_lbm_]^§^	Positive	74	5	71.2	96.9	93.7	84.0
	Negative	30	158				

Exclusion of 8/275 subregions because a follow-up PSMA-PET/CT showed the persistence or progression of the PET-positive lesion(s) indicating that the LN(s) had not been removed at surgery

*PPV* positive predictive value, *NPV* negative predictive value

^†^A PSMA-PET-positive lesion was defined as focal tracer accumulation greater than normal or physiological local background activity

^‡^Cut-off used > 23 counts per second, decay corrected to 48 h and normalised to injected activity megabecquerel per kilogram lean body mass

^§^Cut-off used > 0.276 tracer uptake (percent injected activity per kilogram lean body mass)

Figure 5a shows an overlap of 51.9% (54/104) for true-positive subregions for all three methods in the Venn diagram. With 83/104 true-positive-rated subregions, the detection rate of PET/CT seems to be excellent compared with the germanium detector (75/104) and γ-probe (64/104) measurement. This might result from the fact that the resected mixed-tissue samples (LN, fat, fibrofatty tissue) were larger when undergoing ex situ measurements by germanium detector and γ-probe than the spatial resolution of PET/CT. Under these circumstances, PET/CT is able to discriminate between lesions with specific PSMA uptake and the surrounding tissue with lower, unspecific uptake than the ex situ measurements.

Interestingly, there was no overlap for false-positive subregions between PET/CT, germanium detector and γ-probe (Fig. 5b). There is no obvious reason for this result but the methodology of the three measurements is very different and the uptake times between [^68^Ga]PSMA-11 imaging and [^111^In]PSMA-617 measurements differ too. While PSMA-PET/CT was recorded 1 h post injection, it might have a higher uptake in regions with higher perfusion. [^111^In]PSMA-617 was measured 48 h post injection with nearly complete tracer wash-out but remaining unspecific tracer accumulation [27].

Germanium detector measurements are very accurate in determining activity in specified sample geometry. For larger samples containing mixed-tissue compositions with fat, fibrofatty tissue, small vessels and LN, the interpretation of the results can only be done as an averaged value for the whole sample. In addition to a generally possible lack of PSMA expression, false-negative results might be caused by very low tumour load (e.g. very small LN) and attenuation of tracer signal due to enclosed tissue in the sample (Fig. 5d). Measurements of mixed-tissue samples with a germanium detector as presented in this study are therefore not comparable to the known excellent sensitivity (92.3%) and specificity (93.5%) from the analysis of “single lymph nodes” in such a device [11]. The reasons are the methodological limitations discussed.

As shown by our group previously [35], the LN detection rate for PSMA-PET/CT was reduced to 50% for tumour deposits < 2.2 mm in LNM; consequently, one could assume that the false-negative subregions might harbour very small amounts of tumour (only detectable at histopathology) or tumour cells were negative for PSMA.

However, currently, we could not identify a common pattern of subregions rated as false negative or false positive on PET/CT, γ-probe or at germanium detector analysis. Neither the clinical data from patients providing those subregions nor the locations of the subregions were apparently different from the rest.

The majority of our small cohort developed clinical recurrence (14/23) (follow-up was 31.2 ± SD 12.8 months). From our experience [17] and the latest literature (meta-analysis of 27 series), it is known that the 2- and 5-year biochemical progression-free survival rates range from 23 to 64% and from 6 to 31%, respectively; the 5-year overall survival is approximately 84% [18, 36]. By rough estimation, our cohort has an outcome which is not better than that of patients treated with conventional salvage LND.

Currently, salvage LND for patients with nodal recurrent LNM or nodal high-volume LNM at primary therapy (rad. PE) at our centre is done guided by ^99m^Tc-labelled PSMA with ex situ γ-probe measurement but without germanium detector measurement in the laboratory, similar to the approach of Maurer et al. [25].

The clinical benefit of RGS salvage LND compared with conventional salvage LND is still unclear. Knipper et al. made the first approach of comparing conventional salvage LND (*n* = 29) versus salvage LND with radio-guided assistance (*n* = 13) [31]. After a short follow-up (6 weeks), they were able to show a better PSA decline in general for the RGS salvage LND compared with conventional salvage LND [31]. It can be assumed that the trend towards assisted or guided surgery such as RGS will continue, bearing in mind that more information before surgery and during surgery will potentially achieve better results.

Bimodal-labelled anti-PSMA antibodies such as ^68^Ga-Glu-urea-Lys-HBED-CC-IRDye800CW/^68^Ga-Glu-urea-Lys-(HE)_3_-HBED-CC-IRDye800CW enabling the tracking of tumour tissue via fluorescence and γ-emission during surgery are under investigation [37, 38]. By using fluorescence imaging as a tool to separate healthy LN tissue from LNM during robot-assisted laparoscopy, bimodal-labelled anti-PSMA antibodies are of great interest [39].

### Limitations

Although the number of patients (*n* = 23) seems to be relatively low, the actual sample size (*n* = 864 LN from 275 subregions) was very high. Even if the majority of the patients were at high risk for PCa, heterogeneity was present (PSA level, Gleason score, different kind of previous therapies).

Generally, there is a selection bias because only patients with suspected LNM on PSMA-PET/CT and therefore known PSMA-positive lesions were included in this study. Furthermore, ideally in all patients, an identical bilateral template LA should have been performed which is hard to realise for all patients for different reasons (e.g. surgical access). However in our cohort, only 4/23 (13%) received a unilateral lymphadenectomy (at the site of a PET-positive lesion); this might weaken the reliability of sensitivity and specificity in our calculation. However, our high number of analysed “subregions” (small anatomical regions) (*n* = 275) and our high number of removed LN (*n* = 864) might provide better diagnostic information than a simple summary in, e.g. left- or right-sided lymph nodes. In summary, 179 PET-negative and 88 PET-positive subregions underwent an LA, which allows, in our view, specificity and sensitivity to be calculated.

## Conclusions

At a region-based analysis, the results for tracer uptake measurements (γ-probe, germanium detector) were significantly different between tumour-affected and tumour-free samples. In the clinical setting with the need for high specificity (> 95%) and a corresponding chosen threshold for γ-probe measurement (confirmed by precise germanium detector measurements), the use of [^111^In]PSMA-617 at LA is feasible for the distinction between affected and unaffected tissue samples and valuable for ex situ tumour verification during surgery.

## Supplementary information

**Additional file 1: Table S1.** PSA-response and clinical course after surgery.

## Data Availability

The datasets used and analysed during the current study are available from the corresponding author on reasonable request.

## Abbreviations

%IA_lbm_: Percent injected activity per kilogram lean body mass
CPS: Counts per second
LA: Lymphadenectomy
LN: Lymph node
LNM: Lymph node metastasis
PCa: Prostate cancer
PET/CT: Positron emission tomography/computed tomography
PSA: Prostate-specific antigen
PSMA: Prostate-specific membrane antigen

## References

[1] American Cancer Society A, Georgia (2018). Cancer Facts & Figures 2018.

[2] Leitlinienprogramm Onkologie (Deutsche Krebsgesellschaft DK, AWMF). Interdisziplinäre Leitlinie der Qualität S3 zur Früherkennung, Diagnose und Therapie der verschiedenen Stadien des Prostatakarzinoms, Langversion 5.0. AWMF Registernummer: 043/022OL, http://www.leitlinienprogramm-onkolo-giede/leitlinien/prostatakarzinom/ (abgerufen am: 10052018). 2018.

[3] Cornford P, Bellmunt J, Bolla M, Briers E, De Santis M, Gross T (2017). EAU-ESTRO-SIOG guidelines on prostate cancer. Part II: treatment of relapsing, metastatic, and castration-resistant prostate cancer. Eur Urol..

[4] Mottet N, Bellmunt J, Bolla M, Briers E, Cumberbatch MG, De Santis M (2017). EAU-ESTRO-SIOG guidelines on prostate cancer. Part 1: screening, diagnosis, and local treatment with curative intent. Eur Urol..

[5] Abdollah F, Gandaglia G, Suardi N, Capitanio U, Salonia A, Nini A (2015). More extensive pelvic lymph node dissection improves survival in patients with node-positive prostate cancer. Eur Urol..

[6] Jilg CA, Drendel V, Rischke HC, Beck T, Vach W, Schaal K (2017). Diagnostic accuracy of Ga-68-HBED-CC-PSMA-ligand-PET/CT before salvage lymph node dissection for recurrent prostate cancer. Theranostics..

[7] Herlemann A, Wenter V, Kretschmer A, Thierfelder KM, Bartenstein P, Faber C (2016). (68)Ga-PSMA positron emission tomography/computed tomography provides accurate staging of lymph node regions prior to lymph node dissection in patients with prostate cancer. Eur Urol..

[8] Afshar-Oromieh A, Zechmann CM, Malcher A, Eder M, Eisenhut M, Linhart HG (2014). Comparison of PET imaging with a (68)Ga-labelled PSMA ligand and (18)F-choline-based PET/CT for the diagnosis of recurrent prostate cancer. Eur J Nucl Med Mol Imaging..

[9] Eiber M, Maurer T, Souvatzoglou M, Beer AJ, Ruffani A, Haller B (2015). Evaluation of hybrid (6)(8)Ga-PSMA ligand PET/CT in 248 patients with biochemical recurrence after radical prostatectomy. J Nucl Med..

[10] Maurer T, Gschwend JE, Rauscher I, Souvatzoglou M, Haller B, Weirich G (2016). Diagnostic efficacy of (68)Gallium-PSMA positron emission tomography compared to conventional imaging for lymph node staging of 130 consecutive patients with intermediate to high risk prostate cancer. J Urol..

[11] Mix M, Reichel K, Stoykow C, Bartholoma M, Drendel V, Gourni E, et al. Performance of (111)In-labelled PSMA ligand in patients with nodal metastatic prostate cancer: correlation between tracer uptake and histopathology from lymphadenectomy. Eur J Nucl Med Mol Imaging. 2018. 10.1007/s00259-018-4094-0 30062606

[12] Hope TA, Goodman JZ, Allen IE, Calais J, Fendler WP, Carroll PR (2019). Metaanalysis of (68)Ga-PSMA-11 PET accuracy for the detection of prostate cancer validated by histopathology. J Nucl Med..

[13] Afshar-Oromieh A, Malcher A, Eder M, Eisenhut M, Linhart HG, Hadaschik BA (2013). PET imaging with a [68Ga]gallium-labelled PSMA ligand for the diagnosis of prostate cancer: biodistribution in humans and first evaluation of tumour lesions. Eur J Nucl Med Mol Imaging..

[14] Perera M, Papa N, Roberts M, Williams M, Udovicich C, Vela I, et al. Gallium-68 prostate-specific membrane antigen positron emission tomography in advanced prostate cancer-updated diagnostic utility, sensitivity, specificity, and distribution of prostate-specific membrane antigen-avid lesions: a systematic review and meta-analysis. Eur Urol. 2019. 10.1016/j.eururo.2019.01.049 30773328

[15] Rauscher I, Kronke M, Konig M, Gafita A, Maurer T, Horn T, et al. Matched-pair comparison of (68)Ga-PSMA-11 and (18)F-PSMA-1007 PET/CT: frequency of pitfalls and detection efficacy in biochemical recurrence after radical prostatectomy. J Nucl Med. 2019. 10.2967/jnumed.119.229187 PMC6954457 31253741

[16] Szabo Z, Mena E, Rowe SP, Plyku D, Nidal R, Eisenberger MA (2015). Initial evaluation of [(18)F]DCFPyL for prostate-specific membrane antigen (PSMA)-targeted PET imaging of prostate cancer. Mol Imaging Biol..

[17] Jilg CA, Rischke HC, Reske SN, Henne K, Grosu AL, Weber W (2012). Salvage lymph node dissection with adjuvant radiotherapy for nodal recurrence of prostate cancer. The Journal of urology..

[18] Fossati N, Suardi N, Gandaglia G, Bravi CA, Soligo M, Karnes RJ (2019). Identifying the optimal candidate for salvage lymph node dissection for nodal recurrence of prostate cancer: results from a large, multi-institutional analysis. Eur Urol..

[19] Abdollah F, Briganti A, Montorsi F, Stenzl A, Stief C, Tombal B, et al. Contemporary role of salvage lymphadenectomy in patients with recurrence following radical prostatectomy. European urology. 2014. 10.1016/j.eururo.2014.03.019 24698524

[20] Zschaeck S, Wust P, Beck M, Wlodarczyk W, Kaul D, Rogasch J (2017). Intermediate-term outcome after PSMA-PET guided high-dose radiotherapy of recurrent high-risk prostate cancer patients. Radiat Oncol..

[21] Maurer T, Robu S, Schottelius M, Schwamborn K, Rauscher I, van den Berg NS, et al. (99m)Technetium-based prostate-specific membrane antigen-radioguided surgery in recurrent prostate cancer. Eur Urol. 2018. 10.1016/j.eururo.2018.03.013 29625755

[22] Schottelius M, Wirtz M, Eiber M, Maurer T, Wester HJ (2015). [(111)In]PSMA-I&T: expanding the spectrum of PSMA-I&T applications towards SPECT and radioguided surgery. EJNMMI Res.

[23] Robu S, Schottelius M, Eiber M, Maurer T, Gschwend J, Schwaiger M (2017). Preclinical evaluation and first patient application of 99mTc-PSMA-I&S for SPECT imaging and radioguided surgery in prostate cancer. J Nucl Med..

[24] Gourni E, Canovas C, Goncalves V, Denat F, Meyer PT, Maecke HR (2015). (R)-NODAGA-PSMA: a versatile precursor for radiometal labeling and nuclear imaging of PSMA-positive tumors. PLoS One.

[25] Maurer T, Robu S, Schottelius M, Schwamborn K, Rauscher I, van den Berg NS (2019). (99m)Technetium-based prostate-specific membrane antigen-radioguided surgery in recurrent prostate cancer. Eur Urol..

[26] Horn T, Kronke M, Rauscher I, Haller B, Robu S, Wester HJ (2019). Single lesion on prostate-specific membrane antigen-ligand positron emission tomography and low prostate-specific antigen are prognostic factors for a favorable biochemical response to prostate-specific membrane antigen-targeted radioguided surgery in recurrent prostate cancer. Eur Urol..

[27] Schollhammer R, De Clermont GH, Yacoub M, Quintyn Ranty ML, Barthe N, Vimont D (2019). Comparison of the radiolabeled PSMA-inhibitor (111)In-PSMA-617 and the radiolabeled GRP-R antagonist (111)In-RM2 in primary prostate cancer samples. EJNMMI Res..

[28] Maurer T, Weirich G, Schottelius M, Weineisen M, Frisch B, Okur A (2015). Prostate-specific membrane antigen-radioguided surgery for metastatic lymph nodes in prostate cancer. Eur Urol..

[29] Rauscher I, Duwel C, Wirtz M, Schottelius M, Wester HJ, Schwamborn K (2017). Value of (111) In-prostate-specific membrane antigen (PSMA)-radioguided surgery for salvage lymphadenectomy in recurrent prostate cancer: correlation with histopathology and clinical follow-up. BJU Int..

[30] Rauscher I, Maurer T, Souvatzoglou M, Beer AJ, Vag T, Wirtz M (2016). Intrapatient comparison of 111In-PSMA I&T SPECT/CT and hybrid 68Ga-HBED-CC PSMA PET in patients with early recurrent prostate cancer. Clin Nucl Med.

[31] Knipper S, Tilki D, Mansholt J, Berliner C, Bernreuther C, Steuber T (2019). Metastases-yield and prostate-specific antigen kinetics following salvage lymph node dissection for prostate cancer: a comparison between conventional surgical approach and prostate-specific membrane antigen-radioguided surgery. Eur Urol Focus..

[32] Unterweger MP, Hoppes DD, Schima FJ. New and revised half-life measurements results. Nuclear instruments and methods in physics research section a: accelerators, spectrometers, detectors and associated equipment. 1992;312(1-2):349–52.

[33] Janmahasatian S, Duffull SB, Ash S, Ward LC, Byrne NM, Green B (2005). Quantification of lean bodyweight. Clin Pharmacokinetics..

[34] Dindo D, Demartines N, Clavien PA (2004). Classification of surgical complications: a new proposal with evaluation in a cohort of 6336 patients and results of a survey. Ann Surg..

[35] Jilg CA, Drendel V, Rischke HC, Beck TIR, Reichel K, Kronig M, et al. Detection rate of (18)F-choline-PET/CT and (68)Ga-PSMA-HBED-CC-PET/CT for prostate cancer lymph node metastases with direct link from PET to histopathology: dependence on the size of tumor deposits in lymph nodes. J Nucl Med. 2019. 10.2967/jnumed.118.220541 PMC6604697 30683768

[36] Ploussard G, Gandaglia G, Borgmann H, de Visschere P, Heidegger I, Kretschmer A (2019). Salvage lymph node dissection for nodal recurrent prostate cancer: a systematic review. Eur Urol..

[37] Baranski AC, Schafer M, Bauder-Wust U, Roscher M, Schmidt J, Stenau E, et al. PSMA-11 derived dual-labeled PSMA-inhibitors for preoperative PET imaging and precise fluorescence-guided surgery of prostate cancer. J Nucl Med. 2017. 10.2967/jnumed.117.201293 29191856

[38] Schottelius M, Wurzer A, Wissmiller K, Beck R, Koch M, Gorpas D (2019). Synthesis and preclinical characterization of the PSMA-targeted hybrid tracer PSMA-I&F for nuclear and fluorescence imaging of prostate cancer. J Nucl Med..

[39] Meershoek P, KleinJan GH, van Oosterom MN, Wit EMK, van Willigen DM, Bauwens KP (2018). Multispectral-fluorescence imaging as a tool to separate healthy from disease-related lymphatic anatomy during robot-assisted laparoscopy. J Nucl Med..

