# Supplementary material for: Results from extended lymphadenectomies with [111In]PSMA-617 for intraoperative detection of PSMA-PET/CT-positive nodal metastatic prostate cancer
Source: EJNMMI Res. 2020 Mar 6;10:17. doi: 10.1186/s13550-020-0598-2 (PMC7060305; doi:10.1186/s13550-020-0598-2)
Supplement: Supplementary file 1 — Additional file 1: Table S1. PSA-response and clinical course after surgery. [file 13550_2020_598_MOESM1_ESM.docx]

**Table S1** PSA-response and clinical course after surgery

| N° | **PSA at**  **latest follow-up (ng/ml)** | **Time from surgery to latest follow up (months)** | **PCa specific therapy at latest follow-up** | **Imaging at latest follow up** | **PCa-stage at latest follow-up** |
| --- | --- | --- | --- | --- | --- |
| *1* | 0,03 | 39,5 | None | None | - |
| *2* | 0,01 | 23,5 | CRCP / Chemotherapy | CT-body | Progressive disease |
| *3* | 1,15 | 25,8 | None | PSMA-PET/CT | Previously known stable LNM |
| *4* | 4,85 | 19,3 | None | PSMA-PET/CT | Moderate disease progression |
| *5* | 18,2 | 23,0 | None | CT-body | Moderate disease progression |
| *6* | 0,03 | 19,4 | None | PSMA-PET/CT | Moderate disease progression |
| *7* | 403,7 | 44,1 | Lutetium-177-PMSA-radioligang therapy | PSMA-PET/CT | Progressive disease |
| *8* | 1,4 | 19,4 | ADT | PSMA-PET/CT | Stable disease |
| *9* | 0,55 | 45,4 | None | PSMA-PET/CT | Stable disease |
| *10* | 54,34 | 45,3 | ADT | PSMA-PET/CT | Progressive disease |
| *11* | 4 | 21,7 | ADT | PSMA-PET/CT | Progressive disease |
| *12* | 0,48 | 26,0 | None | None | - |
| *13* | 0,43 | 50,4 | ADT | PSMA-PET/CT | Progressive disease |
| *14* | 5,02 | 47,6 | None | PSMA-PET/CT | Progressive disease |
| *15* | 0,07 | 2,4 | None | None | - |
| *16* | 10,35 | 42,0 | CRCP / Chemotherapy | PSMA-PET/CT | Progressive disease |
| *17* | 4,97 | 40,8 | None | PSMA-PET/CT | Moderate disease progression |
| *18* | 0,7 | 34,0 | None | None | - |
| *19* | 0,03 | 37,0 | ADT | PSMA-PET/CT | Previously known stable LNM |
| *20* | 0,1 | 8,2 | None | MRI pelvic | No results |
| *21* | 1,34 | 32,9 | None | PSMA-PET/CT | Progressive disease |
| *22* | 31,6 | 35,2 | ADT / CRCP | CT-body | Progressive disease |
| *23* | 26,3 | 33,7 | CRCP / Chemotherapy | PSMA-PET/CT | Progressive disease |

LNM = Lymph node metastases

CRPC = castration resistant prostate cancer

ADT = Androgen Deprivation therapy
